# A Cross-Sectional Study of Socio-Demographic Factors and Vaccination Compliance Among Children Under Two in Bhubaneswar, India, With Emphasis on Parental Education

**DOI:** 10.7759/cureus.81610

**Published:** 2025-04-02

**Authors:** Preetinanda Parida, Vishwanath Ghoshal, Mirabai Das, Manas K Nayak, Nirmal K Mohakud

**Affiliations:** 1 Biochemistry, Kalinga Institute of Medical Sciences, Bhubaneswar, IND; 2 Preventive and Social Medicine, Kalinga Institute of Medical Sciences, Bhubaneswar, IND; 3 Preventive and Social Medicine, Teerthanker Mahaveer University, Moradabad, IND; 4 Health and Education, Kalinga Institute of Social Sciences, Bhubaneswar, IND; 5 Pediatrics, Kalinga Institute of Medical Sciences, Bhubaneswar, IND

**Keywords:** developing country, immunization coverage, parental education, rotavirus, socio-demographic factors, universal immunization, vaccination, vaccination compliance, vaccine-preventable diseases

## Abstract

Background: Vaccination remains a cornerstone of child survival, yet coverage in low-resource settings often falls below global targets. Socio-demographic disparities, such as parental education, income, and healthcare access, persistently influence vaccine uptake in developing countries. This study focuses on Bhubaneswar, India, where rapid urbanization intersects with inequities in immunization access, to identify actionable predictors of compliance among children under the age of two.

Objectives: This cross-sectional study aimed to assess the association between socio-demographic factors, including parental education, and vaccination compliance among children under the age of two in Bhubaneswar, a developing country setting.

Methods: A cross-sectional study was done in the pediatric outpatient department (OPD) at a teaching institute. This study was conducted in a teaching hospital in Eastern India from November 2019 to April 2020. The children aged 10 to 24 months attending immunization clinics/pediatrics OPD were selected for the study. The immunization records of enrolled children were collected at the hospital by visiting their homes or using WhatsApp (Meta Platforms, Inc., Menlo Park, California, United States). Demographic and clinical details (age, sex, living area, and maternal/paternal education) were recorded in a predesigned proforma. The data were collected by a trained researcher and entered into a Microsoft Excel (Microsoft Corporation, Redmond, Washington, United States) datasheet.

Results: We enrolled 851 children in this study, which revealed a Bacillus Calmette-Guérin (BCG) vaccine coverage rate of 787 (92.48%), declining with age. Oral polio vaccine (OPV) coverage ranged from 680 (79.91%) at birth to 783 (92.01%) by 14 weeks, while diphtheria, pertussis, and tetanus (DPT) and fractional inactivated polio vaccine (IPV) rates were quite low, ranging from 220 (25.9%) at six weeks to 271 (31.84%) at 14 weeks. Pentavalent vaccine coverage was higher, between 439 (51.6%) at six weeks and 496 (58.3%) at 14 weeks. Only 357 (41.95%) of children under the age of two were fully immunized. Maternal and paternal education significantly influenced vaccine compliance (p < 0.05), with higher education correlating with better coverage. Vaccines outside the state program, such as pneumococcal conjugate vaccine (PCV) and rotavirus, had low uptake. Additionally, there were socio-demographic disparities in vaccine coverage.

Conclusion: This study highlights low vaccination compliance, with only 357 (41.95%) children under two years fully immunized in Bhubaneswar, India. Parental education predicts higher coverage, especially for BCG, DPT, and non-state-sponsored vaccines (hepatitis A and chickenpox). Socio-demographic disparities emphasize the need for targeted interventions integrating education and equitable access to improve immunization outcomes in a developing setup.

## Introduction

Immunization is a cornerstone of child survival, particularly in developing countries like India, where vaccine-preventable diseases (VPDs) remain a significant public health challenge. Despite the protection offered by maternal antibodies and breastfeeding, this innate immunity wanes over time, leaving children vulnerable to life-threatening illnesses such as tuberculosis, measles, and polio [1,2]. India’s Universal Immunization Program (UIP) provides free vaccines against 11 diseases, yet coverage remains suboptimal, with only 60% of children fully immunized as of 2017-18 [3,4]. This gap persists despite initiatives like Mission Indradhanush, which aimed to boost immunization rates [5,6].

Globally, countries like Sri Lanka and Bangladesh have achieved near-universal immunization coverage, highlighting the potential for improvement in India [7,8]. However, disparities in vaccine uptake persist, influenced by socio-demographic factors such as parental education, income, and healthcare access [9,10]. Parental education, in particular, has been shown to significantly impact immunization compliance, as educated parents are more likely to understand the importance of vaccines and adhere to schedules [11,12].

This study focuses on Bhubaneswar, India, to assess immunization coverage among children aged 10-24 months and explore its association with parental education and other socio-demographic factors. By identifying barriers to vaccination, this research aims to inform targeted interventions to improve immunization rates and reduce the burden of VPDs in similar settings.

## Materials and methods

Study design, setting, and duration

This cross-sectional study was conducted at Kalinga Institute of Medical Sciences (KIMS), a tertiary care teaching hospital in Bhubaneswar, Eastern India, from November 2019 to April 2020. The hospital serves a diverse population, including urban, peri-urban, and rural communities, making it an ideal setting for assessing immunization coverage and its determinants. The study duration was six months, ensuring adequate time for data collection and analysis.

Inclusion and exclusion criteria

Children aged 10-24 months attending the immunization clinic or pediatric outpatient department (OPD) at KIMS were included. This age group was selected as it represents a critical cohort for evaluating full vaccination compliance under India’s Universal Immunization Programme (UIP), ensuring the completion of the primary vaccination series.

Children with chronic illnesses or congenital conditions that could interfere with routine immunization schedules, as well as those whose parents/caregivers declined participation, were excluded.

Sampling technique

Systematic sampling was employed to ensure representativeness. Every third child visiting the immunization clinic or pediatric OPD during the study period was included. This method minimized selection bias and ensured a balanced representation of the target population.

Data collection

Demographic and clinical details, including age, sex, residence (urban/rural), and parental education levels, were recorded using a pre-designed, standardized proforma. Immunization records were collected through multiple sources, including hospital records, home visits, and digital platforms such as WhatsApp (Meta Platforms, Inc., Menlo Park, California, United States) for verification. Vaccinations administered under government programs were cross-verified using unique identification numbers on vaccination cards, while private vaccinations were confirmed through parental records or associated clinics. A trained researcher collected and entered the data into Microsoft Excel (Microsoft Corporation, Redmond, Washington, United States) for analysis.

Statistical analysis

Descriptive statistics were used to summarize participant characteristics, such as age, sex, residence, and parental education. Associations between socio-demographic factors (e.g., parental education) and vaccination compliance were analyzed using IBM SPSS Statistics for Windows, Version 21 (Released 2012; IBM Corp., Armonk, New York, United States). Chi-square tests and logistic regression were employed to assess the significance of associations, with a p-value <0.05 considered statistically significant.

Ethical considerations

The study was approved by the Institutional Ethical Committee (IEC) of KIMS (Ref. No.: KIMS/KIIT/IEC/101/18.10.2017). Written informed consent was obtained from parents/caregivers before enrollment. The study adhered to the ethical principles outlined in the Declaration of Helsinki, ensuring confidentiality, voluntary participation, and the right to withdraw at any stage without consequences.

## Results

A total of 851 children were enrolled in this study, with immunization cards available for the majority of participants (801, 94.12%). For the remaining 50 (5.88%) children, vaccination status was determined using the recall method. Among the enrolled participants, 539 (63.34%) were male and 312 (36.66%) were female. The majority of respondents were mothers (610, 71.68%), while fathers provided information for 236 (27.73%) children. In five cases, guardians and relatives gave consent on behalf of the parents. Our analysis found that vaccination rates were slightly higher in urban areas compared to rural areas, though this difference was not statistically significant.

The immunization history revealed that while some participants received individual vaccines such as diphtheria, pertussis, and tetanus (DPT), inactivated polio vaccine (IPV), Haemophilus influenzae type b (HiB), and measles, the majority opted for pentavalent and measles, mumps, and rubella (MMR)/MR vaccines. This variation was influenced by state-sponsored immunization programs and individual preferences. Therefore, we combined the results to evaluate the overall coverage of DPT + pentavalent and measles + MMR1 vaccines, while also presenting the individual vaccine coverage for clarity.

The study found a Bacillus Calmette-Guérin (BCG) coverage rate of 787 (92.48%), with coverage declining as the age of the child increased (Table 1). The coverage for the oral polio vaccine (OPV) showed variability: the zero doses were covered at 680 (79.91%), the first dose at 783 (92.01%), the second dose at 751 (88.25%), and the third dose at 694 (81.55%). The coverage rates for fractional IPV doses were significantly low, with only 271 (31.84%), 208 (24.44%), and 55 (6.46%) of children receiving the first, second, and third doses, respectively.

**Table 1:** Vaccination coverage of individual vaccines among study participants. BCG: Bacillus Calmette-Guérin; OPV: oral polio vaccine; IPV: injectable polio vaccine; HiB: Haemophilus influenzae type b; DPT: diphtheria, tetanus, and pertussis; PCV: pneumococcal conjugate vaccine; MMR: measles, mumps, and rubella

S. No	Name of vaccine	Coverage N (%)
1	BCG	787 (92.48%)
2	OPV Zero	680 (79.91%)
3	OPV 1	783 (92.01%)
4	OPV 2	751 (88.25%)
5	OPV 3	694 (81.55%)
6	IPV 1	271 (31.84%)
7	IPV 2	208 (24.44%)
8	IPV 3	55 (6.46%)
9	HiB 1	50 (5.88%)
10	HiB 2	42 (4.94%)
11	HiB 3	32 (3.76%)
12	Pentavalent 1/ DPT 1	764 (89.78%)
13	Pentavalent 2/ DPT 2	729 (85.66%)
14	Pentavalent 3/ DPT 3	668 (78.50%)
15	Rotavirus 1	416 (48.88%)
16	Rotavirus 2	383 (45.01%)
17	Rotavirus 3	300 (35.25%)
18	PCV 1	124 (14.57%)
19	PCV 2	109 (12.81%)
20	PCV 3	80 (9.40%)
21	Measles/ MMR 1/ MR 1	487 (57.23%)
22	Fully immunized	357 (41.95%)
23	MMR 2	24 (2.82%)
24	Hepatitis A 1	82 (9.64%)
25	Hepatitis A 2	34 (4.00%)
26	Varicella 1	57 (6.70%)
27	Varicella 2	2 (0.24%)

Many guardians chose to administer the pentavalent vaccine instead of DPT, leading to coverage rates of 764 (89.78%), 729 (85.66%), and 668 (78.50%) for the respective doses given at six, 10, and 14 weeks. Notably, 16 parents mixed doses of DPT and pentavalent at six weeks, nine parents at 10 weeks, and five parents at 14 weeks.

Some children received the HiB vaccine separately, but most received it as part of the pentavalent vaccine. The coverage rates for the HiB vaccine when given alone were very low, at 50 (5.88%), 42 (4.94%), and 32 (3.76%) for the first, second, and third doses, respectively. In contrast, the coverage rates for HiB as part of the pentavalent vaccine were higher, at 764 (89.78%), 729 (85.66%), and 668 (78.50%) for the respective doses. Less than 1% of participants reported having received both HiB and pentavalent vaccines separately (Table 1).

Additionally, the pneumococcal conjugate (PCV) and rotavirus (RV) vaccines were administered at six, 10, and 14 weeks of life. The coverage rates for the pneumococcal conjugate vaccine (PCV) were relatively low, with 124 (14.57%), 109 (12.81%), and 80 (9.40%) of children receiving the first, second, and third doses, respectively. Among those who did receive the RV vaccine, Rotavac was the preferred brand, with coverage rates of 331 (38.9%), 308 (36.2%), and 260 (30.6%) at six, 10, and 14 weeks, respectively. Notably, over half of the respondents did not administer the rotavirus vaccine to their children, as evidenced by the low coverage rates for rotavirus 1 (416 (48.88%)), rotavirus 2 (383 (45.01%)), and rotavirus 3 (300 (35.25%)) Additionally, some parents opted for extra vaccines such as hepatitis A (82 (9.64%) for the first dose and 34 (4.00%) for the second dose) and varicella (57 (6.70%) for the first dose and 2 (0.24%) for the second dose), though coverage for these vaccines remained limited.

At nine months, 45.6% of children received the measles vaccine (MV), 8.1% received the measles-rubella vaccine (MR1 or MMR-1), and 3.5% received both. Only 24 children (2.8%) received the MMR booster dose (MMR2) at 16 months. Overall, 41.9% of the children were fully immunized by the age of one year, with fully immunized male children comprising 67.5% of this group.

The study indicated that maternal education played a significant role in vaccine compliance; mothers with higher secondary education or above were more likely to ensure vaccination for their children, particularly for BCG, PCV, and rotavirus (P value < 0.03) (Table 2).

**Table 2:** Association between maternal education and vaccine compliance of the child. BCG: Bacillus Calmette-Guérin; OPV: oral polio vaccine; IPV: injectable polio vaccine; HiB: Haemophilus influenzae type b; DPT: diphtheria, tetanus, and pertussis; PCV: pneumococcal conjugate vaccine; MMR: measles, mumps, and rubella

Vaccine name	Vaccine taken	Education status of the mother		Chi-square value	p-value
		High secondary and above	Secondary and below		
BCG	Yes	490	297	4.76	0.029
	No	31	33		
OPV(at birth)	Yes	430	250	5.77	0.010
	No	91	80		
OPV 1	Yes	486	297	2.96	0.085
	No	35	33		
OPV 2	Yes	465	286	1.30	0.254
	No	56	44		
OPV 3	Yes	434	260	2.73	0.098
	No	87	70		
DPwT 1/Penta 1	Yes	476	288	3.68	0.055
	No	45	42		
DPwT 2/Penta 2	Yes	456	273	3.78	0.050
	No	65	57		
DPwT 3/Penta 3	Yes	424	244	6.62	0.010
	No	97	86		
Measles + MMR/MR	Yes	297	190	0.026	0.870
	No	224	140		
PCV 1	Yes	98	26	19.39	<0.001
	No	423	304		
PCV 2	Yes	86	23	16.45	<0.001
	No	435	307		
PCV 3	Yes	61	19	8.39	0.004
	No	460	311		
IPV 1	Yes	185	86	8.30	0.004
	No	336	244		
IPV 2	Yes	147	61	10.35	0.001
	No	374	269		
IPV 3	Yes	37	18	0.906	0.341
	No	484	312		
Rotavirus 1	Yes	302	114	44.34	0.0001
	No	219	216		
Rotavirus 2	Yes	284	99	49.03	0.0001
	No	237	231		
Rotavirus 3	Yes	227	73	40.72	0.0001
	No	294	257		

Paternal education also significantly influenced immunization compliance for all vaccines except measles/MMR. A significant association between paternal education and vaccine compliance, with higher vaccine uptake observed among children whose fathers had high secondary education and above, compared to those with secondary education and below. P-values indicate statistically significant differences (p < 0.05) for most vaccines, including BCG, OPV series, and PCV, suggesting paternal education influences vaccine compliance. Notably, rotavirus vaccines demonstrated the strongest association (p < 0.001), highlighting the impact of paternal education on adherence to this vaccine series (Table 3).

**Table 3:** Association between paternal education and vaccine compliance of the child. BCG: Bacillus Calmette-Guérin; OPV: oral polio vaccine; IPV: injectable polio vaccine; HiB: Haemophilus influenzae type b; DPT: diphtheria, tetanus, and pertussis; PCV: pneumococcal conjugate vaccine; MMR: measles, mumps, and rubella

Vaccine name	Vaccine taken	Education status of the father		Chi-square value	p-value
		High secondary and above	Secondary and below		
BCG	Yes	662	125	6.34	0.012
	No	46	18		
OPV (at birth)	Yes	574	106	3.57	0.059
	No	134	37		
OPV 1	Yes	658	125	4.94	0.026
	No	50	18		
OPV 2	Yes	634	117	6.85	0.009
	No	74	26		
OPV 3	Yes	585	109	3.24	0.072
	No	123	34		
DPwT 1/Penta 1	Yes	644	120	6.43	0.011
	No	64	23		
DPwT 2/Penta 2	Yes	618	111	9.05	0.003
	No	90	32		
DPwT 3/Penta 3	Yes	568	100	7.47	0.006
	No	14	43		
Measles + MMR/MR	Yes	415	72	3.32	0.068
	No	293	71		
PCV 1	Yes	115	9	9.46	0.002
	No	593	134		
PCV 2	Yes	101	8	8.00	0.005
	No	607	135		
PCV 3	Yes	73	7	4.09	0.043
	No	635	136		
IPV 1	Yes	234	37	2.82	0.093
	No	474	106		
IPV 2	Yes	184	24	5.45	0.019
	No	524	119		
IPV 3	Yes	46	9	0.008	0.928
	No	662	134		
Rotavirus 1	Yes	368	48	16.13	0.0001
	No	340	95		
Rotavirus 2	Yes	343	40	20.14	0.0001
	No	365	103		
Rotavirus 3	Yes	271	29	16.88	0.0001
	No	437	114		

## Discussion

This study revealed several important findings regarding vaccination compliance among children aged 10 to 24 months in a tertiary care setting. Notably, we found that fully immunized male children outnumbered female children (63.34% male vs. 36.66% female), echoing previous research highlighting gender disparities in immunization rates [13]. The preference for male children at birth may contribute to this imbalance, as evidenced by the lower coverage of essential vaccines like BCG, OPV, DPT, and measles in female children compared to their male counterparts [14].

Our analysis indicated that while certain vaccines like PCV, MMR2, typhoid, and hepatitis A are scheduled within the first two years of life, they are not included in the state government's immunization program. Consequently, parents must procure these vaccines at their own expense, which necessitates financial resources and motivation. Maternal education emerged as a significant factor in motivating parents to ensure their children received vaccinations. Some children received multiple vaccines, such as the measles and MMR at nine months, or HiB and pentavalent vaccines within a month at less than 14 weeks of age. This overlap suggests a lack of knowledge among parents regarding the constituents of the vaccines, highlighting the need for better education and awareness [15,16].

We found that maternal education positively influenced compliance with several vaccines, including BCG, OPV, DPT, and rotavirus. This is consistent with findings from Streatfield et al., which indicated that higher-educated women were more likely to obtain immunization information from medical sources than those with less education [17]. Moreover, communication skills linked to maternal education were associated with complete immunization in children, as shown in other studies [15,16]. Similar conclusions have been drawn from various studies indicating that maternal education significantly impacts health-seeking behavior and overall child health outcomes [18,19].

In contrast to many previous studies in India, our research highlights the equal importance of paternal education as a determinant of vaccination compliance. We observed a significant association between fathers' educational attainment and compliance with vaccines such as BCG, OPV, and DPT/Penta. This finding aligns with a study where higher paternal education increased the likelihood of complete immunization [20]. Conversely, factors such as maternal illiteracy and distance from healthcare facilities, identified as barriers in a study in Delhi, also pertain to our findings, emphasizing the need for geographic accessibility to immunization services [21].

Despite a higher initial coverage for vaccines administered in the first month of life, our results indicate a significant decline in coverage for subsequent doses, particularly for DPT and OPV. This trend suggests that vaccination compliance diminishes as children grow older, possibly due to decreased parental focus on vaccinations after the initial months. Our findings align with those of Mathew et al., who reported that vaccination compliance inversely correlates with child age, with compliance rates falling below 50% after the first year [18].

The proportion of fully immunized children in our study was 357 (41.95%), which is significantly lower than the national averages reported in National Family Health Survey (NFHS) 4 (78.6%) and NFHS 3 (51.8%) (Table 4) [22]. This disparity may reflect geographical inequities in healthcare access within our study region compared to national averages, compounded by potential recall bias in vaccination status reporting. Additionally, our cohort included marginalized populations underrepresented in national surveys, likely contributing to lower observed coverage.

**Table 4:** Comparison of a few vaccine indicators observed in this study with NFHS-3 and 4. BCG: Bacillus Calmette-Guérin; DPT: diphtheria, pertussis, and tetanus; NFHS: National Family Health Survey

Indicators	Present study	NFHS 4	NFHS 3
Children aged 12-23 months fully immunized (BCG, measles, and 3 doses each of polio and DPT) (%)	41.9 (N=357)	78.6	51.8
Children aged 12-23 months who have received BCG (%)	92.5 (N=787)	94.1	83.6
Children aged 12-23 months who have received 3 doses of polio vaccine (%)	81.6 (N=694)	82.8	65.1
Children aged 12-23 months who have received 3 doses of DPT vaccine (%)	78.5 (N=668)	89.2	67.9

Although our study found that 92.5% (n=787) of children received BCG at birth, approximately 81.6% (n=694) and 78.5% (n=667) received at least three doses of polio and DPT, respectively. These figures fall within the national averages of NFHS 4 and NFHS 3. Alarmingly, only 57.2% (n=487) of children received the measles vaccine, well below the national average of 87.9% (NFHS 4) [22].

Access to improved drinking water sources among participants (n=619, 72.7%) mirrors national data from NFHS 4 (88.8%) and NFHS 3 (78.4%), suggesting that these families face challenges associated with poor environmental sanitation and socio-economic conditions. Families endure significant economic burdens from preventable infectious diseases like diarrhea, which could be mitigated through enhanced education and immunization, as shown in our previous studies [23,24]. However, our analysis indicates that despite socio-economic disadvantages, children of well-educated parents demonstrate higher compliance with vaccination schedules.

We acknowledge potential confounding factors such as healthcare access disparities, temporal variations in vaccine availability, and unmeasured cultural beliefs that may influence vaccination uptake. While our analysis adjusted for key covariates (e.g., parental education, residence), future longitudinal studies with granular socioeconomic data could better disentangle these complex interactions.

Limitations

While this study provides valuable insights into the influence of parental education on vaccination compliance, there are a few limitations to consider. We have not explicitly highlighted other demographic factors. First, we did not collect data on the geographic location or proximity of healthcare institutions, which could contribute to vaccination compliance. Future studies should include this variable to assess the impact of accessibility to healthcare facilities on immunization rates.

Second, in a small subset of participants, vaccination status was determined using parental recall rather than verified records. This method, while necessary in some cases, may have introduced recall bias. However, the majority (94%) of participants had vaccination cards available, which strengthens the reliability of the data. Future studies could mitigate this limitation by using real-time digital vaccination records or integrating with national immunization databases.

Finally, as a cross-sectional study, our findings demonstrate associations rather than causal relationships between parental education and vaccination compliance. Longitudinal studies would be beneficial to further explore the causality of these associations over time. Despite these limitations, the study offers robust data from a diverse population and highlights critical areas for public health interventions to improve vaccination rates in developing country settings.

## Conclusions

This study highlights suboptimal vaccination compliance (41.95%, fully immunized) among children under two in Bhubaneswar, India, with parental education emerging as a critical predictor of coverage. Higher maternal and paternal education correlated with improved uptake of BCG, DPT, and non-state-sponsored vaccines like PCV and rotavirus. Societal implications underscore the need to address educational disparities and systemic inequities in healthcare access. Strengthening community-based education programs, integrating marginalized populations into immunization drives, and expanding state-sponsored vaccine coverage could bridge these gaps. Prioritizing parental literacy and equitable vaccine distribution will not only reduce preventable child mortality but also foster trust in public health systems, advancing India’s progress toward universal immunization and Sustainable Development Goals (SDGs).
